# Experimental evaluation and surface integrity analysis of cryogenic coolants approaches in the cylindrical plunge grinding

**DOI:** 10.1038/s41598-021-00225-6

**Published:** 2021-10-25

**Authors:** Faruk Abedrabbo, Denis Soriano, Aitor Madariaga, Raúl Fernández, Sepideh Abolghasem, Pedro J. Arrazola

**Affiliations:** 1 Mechanical and Manufacturing Department, Faculty of Engineering, Mondragon Unibertsitatea, 20500 Mondragón, Spain; 2 Department of Industrial Engineering, School of Engineering, Universidad de los Andes, 111711 Bogotá, Colombia

**Keywords:** Engineering, Materials science

## Abstract

Replacement of pollutant fluids with eco-friendly strategies in machining operations significantly contributes to protecting the environment, diminishing global warming, and ensuring a healthier workplace for employees. This study compares cryogenic coolants with conventional coolants in cylindrical plunge grinding using a Cubic Boron Nitride (CBN) wheel. Samples of 27MnCr5 steel used in the manufacture of automotive transmission components were ground using (i) Liquid Nitrogen (LN_2_), (ii) a combination of LN_2_ + Minimum Quantity Lubrication (MQL), and (iii) a conventional coolant. The effects of the different cooling methods on the surface integrity of the ground surfaces were examined in terms of surface roughness, microstructural defects, microhardness profiles, and residual stresses. In general, surface roughness was similar for the tested cooling systems, even after grinding three subsequent surfaces in which the process stability was analyzed. Interestingly, the use of eco-friendly cryogenic systems induced fewer microstructural defects than conventional systems, and particularly, LN_2_+MQL lead to more compressive surface residual stresses that would improve the in-service performance of components. These results show opportunities for replacing conventional pollutant systems with eco-friendly cryogenic strategies for refrigerating/lubricating grinding processes to reduce harmful effects on the environment and pose health risks to operators.

## Introduction

Grinding is a finishing procedure used to achieve good dimensional accuracy and low roughness in mechanical components^1^, especially for materials with challenging machining, like maraging steels, used in the automotive sector for powertrains^2^ or aeronautics and astronautics applications like inertial navigation elements^3^. For this reason, grinding processes make up 25% of total machining operations^4^. The cost of finishing operations such as grinding is nearly 30% of the total manufacturing cost in a mechanical piece^1^. More importantly, from this 30%, between 7 and 17% of the cost is caused by coolant systems^5^. Traditional coolant systems are based on water-miscible cutting fluids, and their high spatial volume requires large storage capacity and high-powered pumps, which further increase the grinding costs^6^. In addition to this impact on the bottom line, coolant systems have a harmful effect on the environment and pose health risks to the operator. According to Hannu et al.^7^, 80% of occupational medical issues correspond to the operator’ contact with water-miscible coolant/lubricant fluids. Respiratory problems and skin diseases caused by contact with cutting fluids are the most common illnesses reported by machine operators^8^.

To reduce these environmental, health, and cost issues in grinding operations, several approaches have been explored. For example, Minimum Quantity Lubricant (MQL) systems reduce the quantity of cutting oil used in the process by controlling the volume sprayed into the machining zone^9^. Other examples of eco-friendly solutions are the use of CO_2_ and LN_2_, in which some authors have found that the cryo-advantages of these solutions reduce temperature and forces in the grinding process without leaving contaminant fluids that are difficult to recycle^10^. For these reasons, cryogenic fluids applied to grinding processes are considered clean technologies^11^. Also, nowadays, the uses of Graphene Oxide (GO) nanosheets water-dispersible have been studied as a potential lubricant in abrasive processes^12^.

Within this context, eco-friendly grinding has become more widespread over time because of stricter government environmental regulations to reduce global warming; these regulations have resulted in a significant increase in the study of cryogenic cooling. Table 1 summarizes the most pertinent literature on coolant/lubricant fluids, materials, properties, and surface integrity parameters studied for cryo-grinding processes over the last 40 years.

**Table 1:** Literature review in cryogenic grinding processes.

Grinding type	Coolant	Material	References	Microstructure	Temperature	Forces	Specific energy	Power	G-ratio	Roughness	Residual stress	Hardness
Surface	Dry, WET, LN_2_	Mild steel AISI 4340 HSS	Chattopadhyay et al.^13^	*		*				*		
		AISI 1020 AISI 1080 AISI D2 AISI H11 AISI M2	Paul et al.^14^	*		*	*			*		
			Paul and Chattopadhyay^15^	*	*	*					*	
			Paul and Chattopadhyay^16^		*	*	*				*	
			Paul and Chattopadhyay^17^		*							
		AISI 304	Ben Fredj and Sidhom^18^	*							*	*
		AISI 1045	Nguyen et al.^19^	*							*	*
		AISI D2	Fathallah et al.^20^	*		*				*	*	*
		Ni-based superalloy	Li et al.^10^			*	*			*	*	
		AISI 1020	Bhaduri et al.^21^			*				*		
		AISI 316	Manimaran et al.^11^	*	*	*	*			*		*
		EN 31	Manimaran et al.^22^	*	*	*				*		
		Ti–6Al–4V	Setti et al.^23^	*		*				*		
		AISI 52100	Reddy et al.^24^					*	*			
		AISI D3	Manimaran et al.^25^	*						*		
		Ti–6Al–4V	Elanchezhian et al.^26^	*	*	*	*			*		
		AISI 52100	Reddy et al.^27^	*				*	*	*	*	
	WET, MQL+CO_2_	X53CrMnNi219 AISI D2	Sanchez et al.^28^		*	*			*	*		*
		GGG70 cast iron	Alberdi et al.^29^			*	*			*		
	Dry, MQL, LN_2_+MQL	Inconel 751	Balan et al.^30^	*	*	*	*			*		
	Dry, Wet, MQL, LN_2_	Inconel 718	Sinha et al.^6^	*		*				*		
	WET, CO_2_	Ti–6Al–4V	Elanchezhian et al.^31^	*	*	*				*		
Cylindrical	WET, MQL+CO_2_	100Cr6	García Gil et al.^32^				*		*			
		52100 18CrMo4	Garcia et al.^33^				*		*		*	
	Cold air jet	BS 970-EN 26	Nguyen et al.^34^		*							*

For several decades, LN_2_ has been applied in many ways, Chattopadhyay et al.^13^ were the first authors that introduced the use of LN_2_ into the surface grinding process in 1985. These authors concluded that forces were diminished, and surface tensile residual stresses could be reduced compared to dry grinding process (DRY) or soluble oil as coolant fluid (WET) when grinding several steel plates. In addition, they determined that under specific conditions, surface roughness was lower when using LN_2_ than WET. About one decade later, the same authors^15,17^ confirmed the hypothesis that LN_2_ reduces forces and induces less tensile residual stresses in surface grinding processes. In these studies, the effects of temperature reduction and microstructure changes were analyzed on the workpiece surfaces and chips for several steel plates. Later in 2014, Setti et al.^23^ studied the influence of cryogenic grinding by comparing the LN_2_, DRY, and WET medium on the surface integrity of titanium. The authors concluded that cooling with a mist of LN_2_ improved the friction behavior at the contact between the workpiece surface and the grinding wheel, reducing the tangential force. Unfortunately, they found that cryogenic conditions at lower speeds produced more defects on the surface workpiece than WET and DRY; however, this effect disappeared at high cutting speeds, where cryogenic conditions generated fewer surface defects than the other coolants.

In 2010, a hybrid MQL+CO_2_ grinding technology was evaluated by Sanchez et al.^28^. This study focused on improving the surface quality, reducing the wear of grinding wheel, and diminishing the lubricant consumption. The results revealed that the mix of CO_2_+MQL produced considerable improvements in the grinding wheel life. Nonetheless, Ra roughness and forces did not present significant differences in comparison with conventional grinding processes. Four years later, Sinha et al.^6^ compared the use of MQL and LN_2_ environment to dry grinding of Inconel 718. They found that using MQL and LN_2_ reduced cutting forces and promoted better grit-workpiece interaction.

In the analysis of the studies summarized in the Table 1, two main tendencies can be highlighted. First, the vast majority of the studies have considered the surface grinding process. Second, the evaluation of improvements resulting from the use of cryo-environmental grinding fluids has been through comparisons of process parameters and the final surface integrity of the workpieces. Given this, the literature on surface integrity lacks investigations on residual stresses and microhardness characterization for cryo-grinding processes. Similarly, surface roughness, forces, and microstructure characterization were the indicators more studied for surface grinding. However, this is not the case of cylindrical plunge grinding, which has several applications in the automotive and machine tool sector but remains relatively understudied. The few works found to have explored this phenomenon were focused only on studying the specific energy of the process and evaluating the wear of the grinding wheel (R-ratio) under several machining conditions. Moreover, no information was found about the use of LN_2_ or LN_2_+MQL in cylindrical grinding as an alternative to reduce pollution of coolant/lubricants fluids, and no study has presented process analysis or surface integrity analysis in terms of forces, power, microstructure, and roughness.

Hence, the present paper investigates options for replacing conventional coolant systems with eco-friendly systems in the plunge grinding. To this end, samples of carburized 27MnCr5 were machined by means of cylindrical plunge grinding with LN_2_ and LN_2_+MQL coolants. The experimentation focused on reducing surface roughness and improving the stability of the process by changing machining conditions. In addition, surface integrity was evaluated in terms of microstructural defects, microhardness profiles, and residual stresses to ensure good surface integrity on the tested material.

## Materials and methods

To analyze the effects of cryogenic coolants on the surface integrity of cylindrical ground pieces, the 27MnCr5 steel commonly used for automotive applications was selected. This material is widely used for gears and shafts due to its high-end hardenability and surface wear resistance. The chemical composition of this ferrite-pearlite steel is given in Table 2.

**Table 2:** Weight percentage chemical composition for the 27MnCr5.

Steel	C	Mn	Si	Cr	B	Ti	Cu	S	P	Al
27MnCr5	0.29	1.21	0.27	1.09	0.0002	0.002	0.19	0.028	0.015	0.023

**Figure 1:**
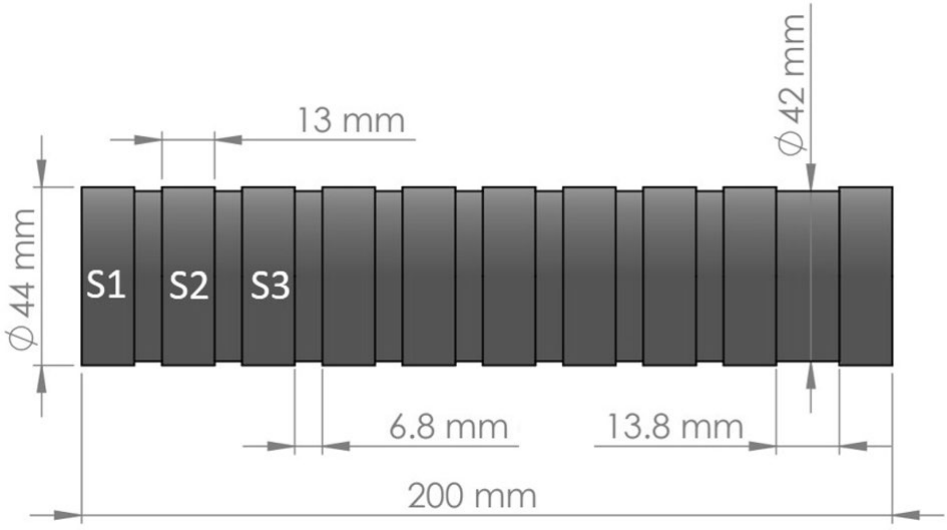
Workpiece scheme and dimensions.

The material was carburized, resulting in a martensite structure with a surface hardness of 59–64 HRC and a carburized depth of 0.7 to 0.9 µm. The material specimens had cylindrical surfaces with a diameter of 44 mm and a width of 13 mm with grooves to separate the surface to be tested. These grooves were 42 mm in diameter and a width of 6.80 mm. The last groove had the same diameter but a width of 13.8 mm. This geometry was designed to identify the side of the specimen and the order of the surfaces to be machined. Figure 1 shows the scheme of this workpiece, in which the surface numbers are shown.

**Figure 2:**
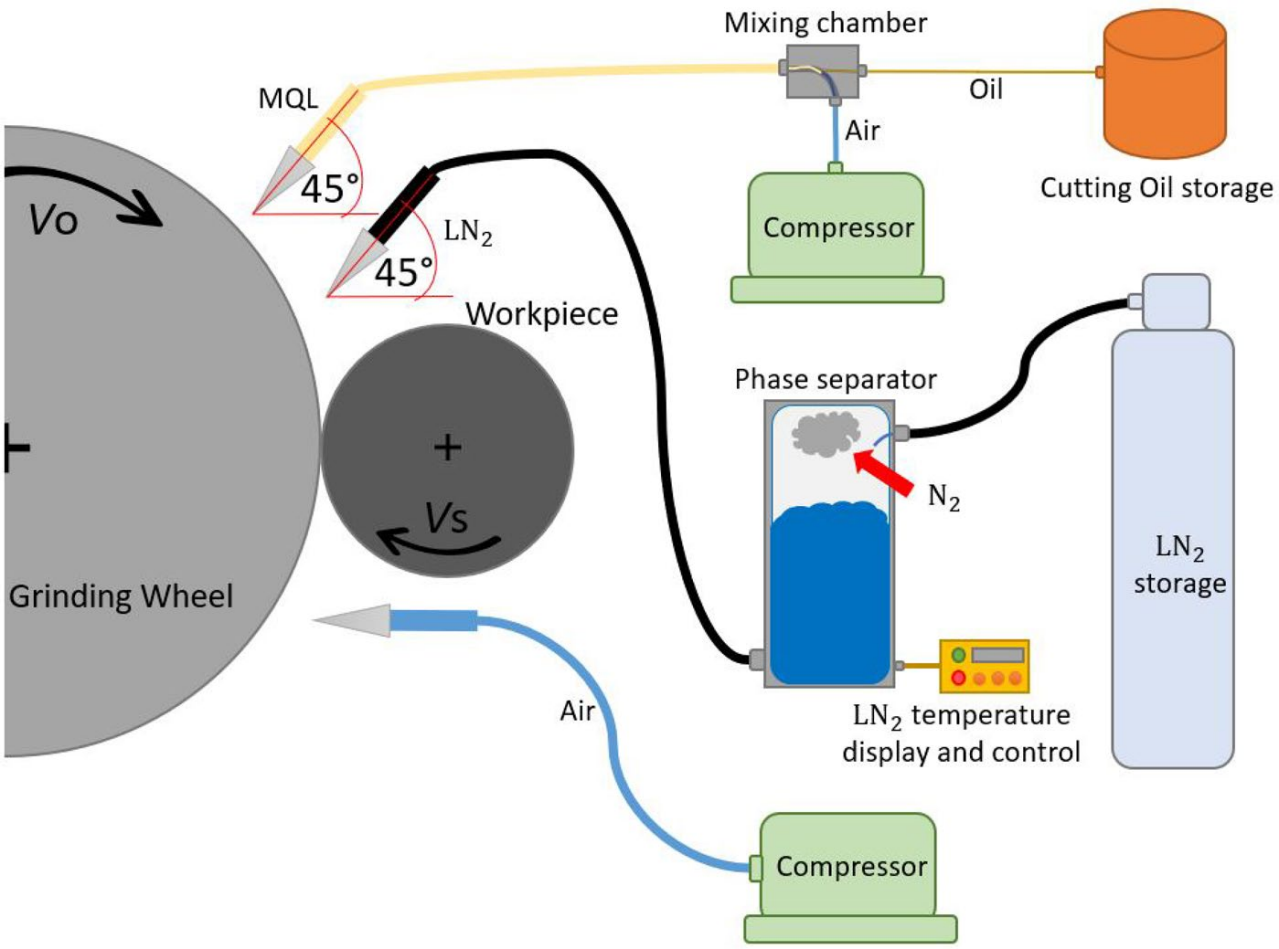
Scheme of the experimental set-up.

The machine used in the experimentation of this work was a GER CU-1000 CNC cylindrical grinding machine, in which the specimens were clamped between centers with a drag claw. Figure 2 describes the set-up used to adapt all coolant/lubricant systems to the machine. The cryogenic coolant system used in this work consisted of a jet of liquid nitrogen (LN_2_), applied directly to the grinding wheel into a near zone between the contact of the wheel and workpiece (2 cm approx.). The LN_2_ system included a liquid storage tank with a pressure of 2 bar. A conduit allowed the nitrogen to fluid from the storage to a phase separator. At the bottom of this phase separator, another conduit allowed LN_2_ to flow to a nozzle while the N_2_ gas remained in the separator’s capsule. This phase separator was required to ensure that the nitrogen was in a liquid stage when it impacted the grinding wheel. At the tip, the LN_2_ system had a circular nozzle of 5 mm in diameter, and the LN_2_ nozzle was set approximately at an angle of 45°. Additionally, an MQL system was used in this work. The MQL consisted of a jet of pressurized air (6–7 bar) with cutting oil particles. With a constant flow of 80 ml/h, this jet was supplied directly to the grinding wheel before the LN_2_ jet (see Fig. 2). Moreover, when the LN_2_, the MQL, or both systems were active, a nozzle of pressurized air (6–7 bar) was applied directly to the grinding wheel below the contact wheel-workpiece to blow away the material particles embedded in the grinding wheel. The third coolant system used in this work was a conventional coolant (WET), compounded by a synthetic water-based emulsion with a concentration of 7.5%, applied directly at the contact between the grinding wheel and workpiece. This liquid was used by the original pumps and pipes of the grinding machine.

The experimentation consisted of carrying out plunge grindings to reduce each surface diameter from 44 to 43.75 mm (224 mm^3^ was ground/removed per surface). In this operation, the effects of machining conditions, coolant systems, and the total ground volume were analyzed on the material’s surface integrity and process stability. For this evaluation, a maximum Ra roughness of 0.32 µm was defined as a limit for good surface quality. In total, eighteen different combinations were tested, and three surfaces were ground subsequently for each combination without dressing the grinding wheel. Three grinding wheel speeds (*V*s) were tested (see Table 3). These speeds were combined with two different workpiece speeds (*V*o) and three different coolant/lubricant systems. A single infeed rate and a sparkout of 10 workpiece revolutions were utilized for all the experiments. A CBN grinding wheel with a diameter of 400 mm and a width of 15 mm was used for the experimental procedure. The reference of this grinding wheel is shown in Table 3. Before testing the three runs of a particular grinding condition, the grinding wheel was dressed to ensure the same surface quality of the wheel for all tested combinations. The dressing depth was 4 µm through 20 passes with a transversal feed of 200 mm/min. The dressing speed changes according to the experiments, taking the same value as *V*s in each experiment.

**Table 3:** Experimental conditions for cylindrical plunge grinding.

Grinding wheel speed (m/s)	40–52.6–63
Workpiece speed (m/min)	13.82–41.47
Infeed rate (mm/min)	0.3
Sparkout (workpiece revolution)	10
Coolant/lubricant systems	WET, LN_2_, LN_2_+MQL
Grinding wheel reference	3D1V-400-15-5-25-27-B107-SA-100-V69M
Grinding machine	GER CU-1000 CNC cylindrical grinding

The surface integrity generated by different plunge grinding conditions was assessed. In a first stage, surface roughness was measured by contact with a Mitutoyo roughness tester SJ-210, following the ISO 4287:1997 standard. The measure had a cut-off length of 0.8 mm and a sampling number of 5, obtaining a total length of 4 mm. The roughness measurements were taken at three equidistant points separated by 120° in the hoop direction, and the mean values of the three tested surfaces were averaged to ensure the quality of this work. On a second stage, the first and the third surfaces of the tested surfaces with lower roughness and better process stability were selected to analyze their surface integrity in greater detail. In this stage, polished ground surfaces were etched with Nital (5%) solution, and the subsurface microstructure was observed on an optical microscope equipped with a digital camera Leica DMC 2900. The micro-hardness profile HV0.05 was examined on the cross-section of specimens with a hardness tester ZWICK and a loading time of 10 s. These profiles were generated from the surface to the bulk of the material in four steps of 25 µm near the surface, followed by four steps of 50 µm, seven steps of 100 µm, and a final increment of 500 µm. Three repetitions per sample were performed. To finish the characterization, residual stresses were measured in the axial and circumferential direction of the ground surfaces by X-ray diffraction technique^35^ employing a portable diffractometer (PROTO iXRD). Three repetitions were conducted to determine the residual stresses of each surface. The radiation employed was a CrKα (λ = 2.291 Å), with a voltage of 25 kV and a current of 5 mA. The (2 1 1) diffraction plane of the martensitic structure was selected for the measurements. Surface residual stresses were measured using a round collimator (1 mm diameter) on the incident beam. Measurements were carried out in Ω mode with 7 inclinations (varying Psi angle from − 37° to 37°) for both axial and circumferential directions. Experimental data was analyzed using PROTO XrdWin software to quantify residual stresses. For this purpose, diffracted peaks were fitted by a Gaussian model, and the diffraction elastic constants used in the calculations were the following: − S_1_ = 1.2 × 10^–6^ MPa^−1^, ½ S_2_ = 6.04 × 10^–6^ MPa^−1^.

## Results and discussion

The detailed surface integrity analysis on surface roughness, microstructural defects, microhardness profiles, and residual stresses is described and discussed in the following subsections.

### Roughness surface analysis

**Figure 3:**
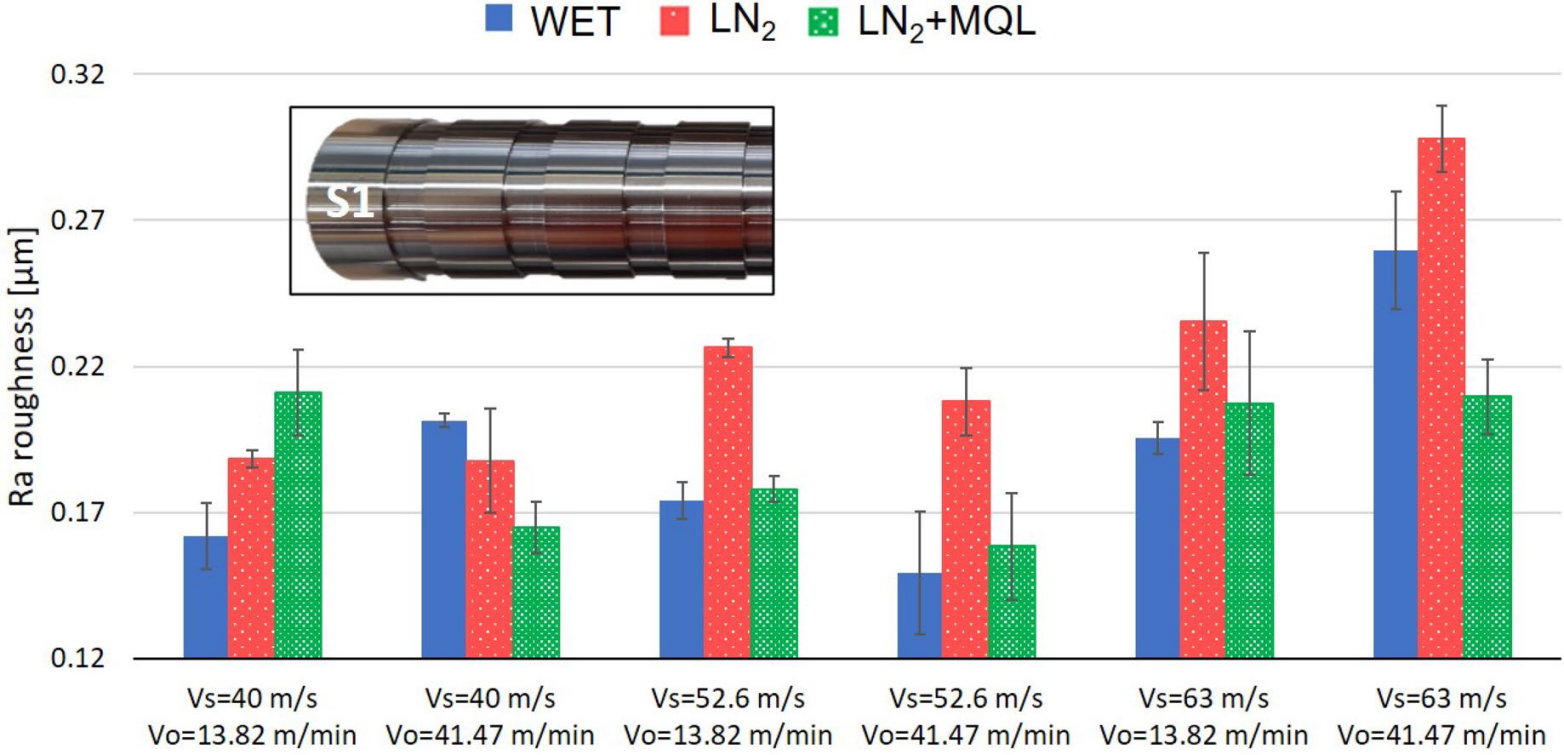
Surface roughness of first ground surfaces.

By evaluating only the surfaces in which the grinding wheel state was the optimum for all tested conditions (after dressing the wheel, i.e., first tested surfaces “S1”), the coolants’ behavior can be summarized in Fig. 3. WET and LN_2_+MQL coolants seem to create better surface quality under specific parameters. In the set of parameters (*V*s = 52.6 m/s, *V*o = 41.47 m/min), WET presented a mean Ra roughness of 0.149 ± 0.013 µm. This value was in agreement with the LN_2_+MQL result, in which the roughness was 0.158 ± 0.014 µm. In contrast, the effect of the LN_2_ system might be different for these grinding parameters. The LN_2_ results showed a high value of roughness (0.208 ± 0.012 µm), placing this liquid in the worst position of the scale because the roughness obtained was the highest under this machining condition. However, this trend seems to change according to the input parameters. For example, the LN_2_ system created a lower surface roughness than WET in the set of parameters (*V*s = 40 m/s, *V*o = 41.47 m/min) with a final roughness of 0.188 ± 0.014; however, this roughness was higher than the LN_2_+MQL. Furthermore, for the set of parameters (*V*s = 40 m/s, *V*o = 13.82 m/min), the LN_2_ produced higher roughness than WET but lower than the LN_2_+MQL with a final roughness of 0.188 ± 0.003 µm. According to these results, for a wheel speed *V*s = 40 m/s, the LN_2_ system seems to produce similar surface roughness independent of the workpiece speed value.

Therefore, the cryogenic grinding with LN_2_ might be recommended to use at low wheel speeds (*V*s = 40 m/s) in order to create the best surface quality. The physical properties of these fluids also support this evidence since the WET and LN_2_+MQL are fluids with two main functions: refrigeration of the grinding wheel and lubrication of the grits on the wheel, allowing the possibility to increase process speed by reducing friction between workpiece-wheel contact area. In contrast, in the LN_2_, when the fluid impacts the wheel, an evaporation process occurs, achieving the function of cooling the grits of the wheel. Still, there is no possibility to lubricate the joint workpiece-wheel. This phenomenon can lead to restricting the use of LN_2_ for higher grinding speeds.

It is crucial to notice in Fig. 3 that uniquely for the condition (*V*s = 40 m/s, *V*o = 41.47 m/min), the LN_2_ and the LN_2_+MQL produced roughness values considerably lower than WET. In the rest of the tested conditions, the LN_2_ might generate roughness values higher than WET, and no specific behavior or tendency was evident concerning the workpiece wheel (Vo). In contrast, for the LN_2_+MQL, the behavior may be different because a slight tendency to decrease roughness was identified when higher workpiece speeds were used regardless of the grinding wheel’s speed. According to Fig. 3, it is also evident that the wheel speed of 63 m/s (regardless of the coolant system and the workpiece speed) produced the worst surface quality of the ground material. This remark suggests that very high wheel speed in the plunge grinding process is highly aggressive to the material, increasing the vibrations of the machine utilized and producing an increased roughness on the surface. Probably, to achieve better surface roughness at higher speeds (< 52.6 m/s), a more robust machine than the one used in this work might be utilized that helps to avoid vibrations during the grinding process.

**Figure 4:**
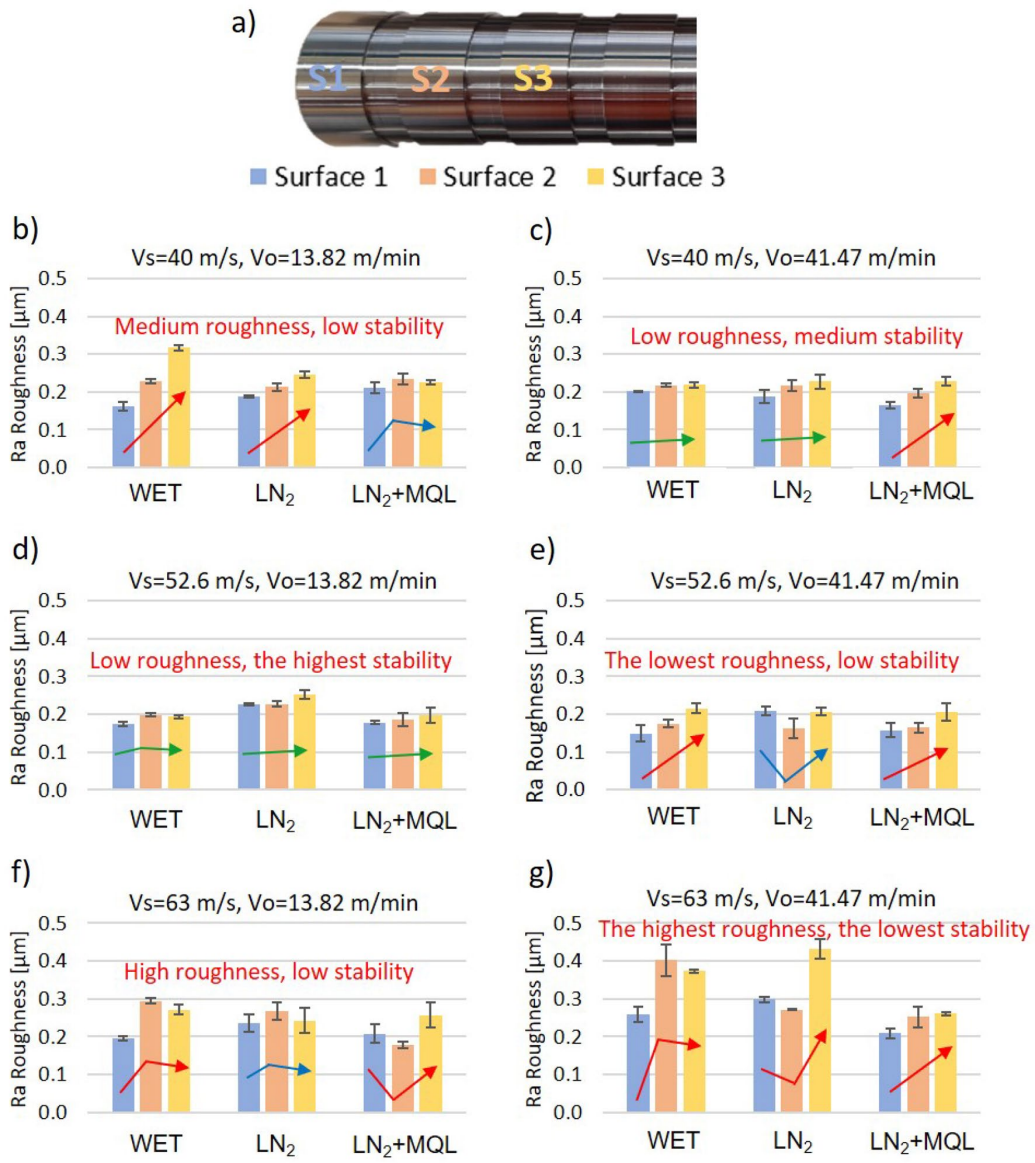
Surface roughness and process stability (identified by arrows) for three subsequent ground surfaces varying input variables.

Several authors have demonstrated that surface roughness on the material increases after several ground pieces without dressing the wheel due to the wear accumulated^36,37^. Thus, this research also analyzed the process stability on surface roughness due to input parameters and the performance of the grinding wheel. Process stability was defined as roughness variation between three subsequent ground surfaces, in which 224 mm^3^ was ground per surface (672 mm^3^ in total, a considerable volume to see the influence of the CBN wheel wear). Considering the total ground volume machined with the grinding wheel, the input process parameters may be classified into two categories: (i) state of surface roughness and (ii) state of process stability. This classification is evident in Fig. 4, which shows the evolution of surface roughness and process stability after three subsequent ground surfaces for each tested set of parameters. Figure 4a shows the subsequent tested surfaces to understand this analysis. Figure 4b displays the first machining condition (*V*s = 40 m/s, *V*o = 13.82 m/min), in which medium values of roughness are found and low stability of the process is presented. Figure 4c shows a decrease in surface roughness (better state, under 0.32 µm) and an improvement in the process's stability (medium stability). The machining condition in this figure (*V*s = 40 m/s, *V*o = 41.47 m/min) improved the process due to increased workpiece speed. This improvement is clear for the WET and LN_2_ coolant systems because of their stability with low slopes. In contrast, this machining condition used with the LN_2_+MQL coolant might show a bad accuracy because of its high slope in process stability.

When the wheel speed of the grinding process increased to 52.6 m/s, the surface quality seems to be better because the surface roughness decreased, and the stability of the process also improved. This effect is shown in Fig. 4d,e for the conditions *V*s = 52.6 m/s with *V*o = 13.82 m/min and *V*s = 52.6 m/s with *V*o = 41.47 m/min respectively. The input parameters presented in these figures induced the lowest values of surface roughness (≈ 0.15 to 0.22 µm). However, in terms of stability, by comparing Fig. 4d with Fig. 4e, the set machining conditions used in Fig. 4e produced low stability in the grinding process. In contrast, the input parameters used in Fig. 4d produced the highest stability on the process for all tested coolant environments. This collection of achieved benefits (lower roughness and stability in the process) enables exceptional accuracy on the final surface obtained by the plunge grinding process, and it improves the productivity of the process by controlling the influence of grinding wheel wear due to not much aggressive contact in the join wheel-workpiece.

The worst results for surface roughness (Ra higher than the limit of 0.32 µm) and process stability, regardless of the coolant system, were found in the grinding conditions: *V*s = 63 m/s with *V*o = 13.82 m/min (Fig. 4f) and *V*s = 63 m/s with *V*o = 41.47 m/min (Fig. 4g). As explained before, the use of a high wheel speed (*V*s = 63 m/s) is highly aggressive for the plunge grinding, producing an increase in surface roughness due to the increase of process vibration. The trend of surface roughness was practically unstable, i.e., it was impossible to detect similar trends in Fig. 4f,g for any coolant conditions. This process instability may be responsible for increasing wheel wear, consequently reducing the productivity cycle of the grinding process. The only remark in these figures is that high workpiece speed (*V*o = 41.47 m/min) further increases the surface roughness for the tested surfaces. This effect was more evident for the LN_2_ samples, in which, for the third ground surface, the value of Ra roughness reached the value of 0.432 ± 0.026 µm.

### Microstructural analysis

**Figure 5:**
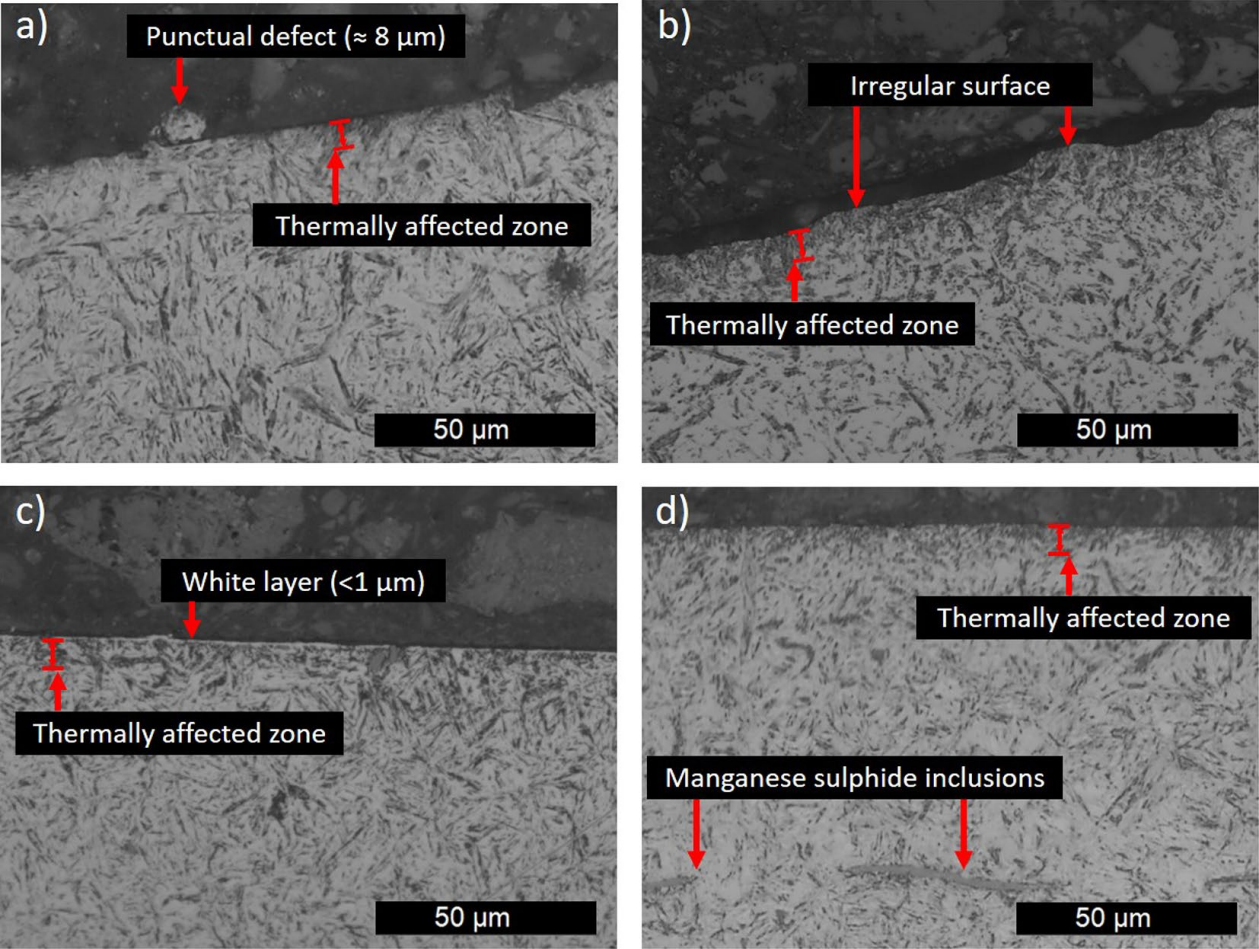
Summary of surface microstructure analysis: (**a**) punctual defect, (**b**) irregular surface, (**c**) white layer, and (**d**) surface without defects.

The ground samples in which surface roughness was lower than 0.32 µm were analyzed for subsurface microstructural changes or defects for both axial and circumferential directions. The analysis is summarized by the photomicrographs shown in Fig. 5. These photomicrographs evidence the types of defects found in the ground surfaces due to the applications of different coolant systems for the plunge grinding process. Since the grinding process tested in this study was carried out in finishing conditions and thus not aggressive conditions, the size of most of the defects detected was less than 4 µm. Therefore, these defects would not affect the material's surface properties.

Figure 5a shows the only significant defect detected for all tested surfaces. This punctual defect was pull-out generated when the WET system was active for the condition (*V*s = 52.6 m/s and *V*o = 13.82 m/min). This pull-out measured approximately 8 µm in diameter, and it probably was generated due to a grit of the grinding wheel breaking and became embedded in the material, causing the generation of a slight micro-chip over the surface. According to Table 4, other punctual defects (pull-outs and gauges) were found on three different machining conditions with sizes around 2 µm.

**Table 4:** Maximum size of microstructural defects. (Empty spaces indicate that the type of defect was not found in that grinding conditions).

Grinding condition				Axial direction				Circumferential direction			
*V*s (m/s)	*V*o (m/s)	Coolant type	Ground surface	Punctual gouge (µm)	White layer (µm)	Irregular surface (µm)	Thermally affected layer (µm)	Punctual gouge (µm)	White layer (µm)	Irregular surface (µm)	Thermally affected layer (µm)
52.6	13.82	WET	S1	2.11	–	3.16	16.64	8.11	1.91	–	13.08
52.6	13.82	LN_2_	S1	–	–	–	16.43	–	–	4.01	13.55
52.6	13.82	LN_2_ + MQL	S1	1.95	–	1.76	22.95	1.96	–	–	20.04
52.6	13.82	WET	S3	–	–	3.25	11.5	–	–	–	15.72
52.6	13.82	LN_2_	S3	–	–	–	12.25	–	–	–	12.6
52.6	13.82	LN_2_ + MQL	S3	–	–	–	12.72	–	–	–	10.32
40	41.47	WET	S1	2.94	–	–	14.72	–	–	2.82	12.71
40	41.47	LN_2_	S1	–	–	–	12.71	–	–	–	10.88
40	41.47	LN_2_ + MQL	S1	–	–	–	10.29	–	–	1.06	11.93
40	41.47	WET	S3	–	–	–	12.05	–	–	–	8.79
40	41.47	LN_2_	S3	–	–	–	11.38	–	–	–	8.36
40	41.47	LN_2_ + MQL	S3	–	–	–	15.38	–	–	–	12.1
52.6	41.47	LN_2_	S1	–	–	–	12.71	-	1.29	–	10.9
52.6	41.47	LN_2_ + MQL	S1	–	–	–	10.04	–	–	–	9.81
52.6	41.47	LN_2_	S3	–	–	–	6.25	–	–	–	8.41
52.6	41.47	LN_2_ + MQL	S3	–	–	–	12.7	–	–	–	8.34

Furthermore, Fig. 5b shows an example of the irregular surface detected on the experimental condition (*V*s = 52.6 m/s and *V*o = 13.82 m/min) when the LN_2_ was applied. This irregular surface measured around 3 to 4 µm. Irregular surfaces were also found for the experimental condition (*V*s = 40 m/s and *V*o = 41.47 m/min) when the WET and LN_2_+MQL systems were applied (Table 4). Additionally, white layers were detected in the ground surfaces generated by a medium wheel speed (*V*s = 52.6 m/s) with thicknesses between 1 and 2 µm (see Fig. 5c).

In all photomicrographs of Fig. 5, the thermally affected layer is clearly defined. This zone is a martensite grain size refinement due to the friction heat between the grits of the grinding wheel and the material surface. Some authors call this zone as Grinding-Induced Layer (GIL)^20,41^. In several cases^20^, the use of LN_2_ decreased the thermally affected layer compared with other coolant methods because the LN_2_ increased the cooling rate in the process. However, this claim is not similar in this research due to several remarks. First, the material explored in this research was carburized. Thus, the martensite refinement might have had less impact on the material microstructure because, after the carburization, the microstructure already presented a martensite phase. Second, the differences in the process might change the effect of the thermally affected layer. Third, according to the data reported in Table 4, all tested conditions showed a thermally affected zone. This thermally affected zone seems to present slight variations between machining conditions, but probably these variations were the uncertainty of the measures. The thermally affected layer did not present trends of evident size reductions between cryogenic coolants and conventional coolants.

Figure 5d shows an example of a ground surface without any defects on the subsurface. As Table 4 illustrates, few conditions produce defects of the ground surface, and the major quantity of defects found was when the WET coolant was applied. In addition, some punctual defects were found for LN_2_ and LN_2_+MQL. Therefore, it seems that cryogenic coolant produces less microstructural defects than WET coolant in the tested conditions.

According to Malkin and Guo^40^, due to the high cutting speed and large strains in grinding processes, the chip-formation is a swift process that can be considered a quasi-adiabatic process because there is no time to transfer the heat generated to the ambient. In WET coolants, the flooding liquid helps to transfer the heat generated in the process to the applied medium^22^ for creating refrigeration to the workpiece. Additionally, WET coolants work as a lubricant between workpiece and grits. However, it is complex for WET fluids to create a liquid film between the wheel-grits and the workpiece, and in some cases, the fluid does not reach the center of the machined surface, limiting the refrigeration effect and promoting the generation of defects in the workpiece surface.

In contrast, despite the cryo-cooling is not a flood cooling and does not achieve the same lubricant function, when the LN_2_ impacts the tool, a mix of liquid and gas cushion produce a freezer effect on the wheel and the workpiece^41^, producing a rapid decrease in the temperature of the process that improves the refrigeration of the wheel and workpiece. This cryogenic cushion reduces the adherence in the joint of grits-workpiece^11^, achieving good refrigeration and reducing surface defects on the material. Furthermore, in MQL, adding oil particles to the LN_2_, the cryogenic cushion between the grinding wheel and workpiece produces both the refrigerating and the lubricating effect. These effects support the result of Table 4, in which the cryogenic strategies show fewer surface defects and might create better surface quality in the machined material.

### Microhardness profiles analysis

**Figure 6:**
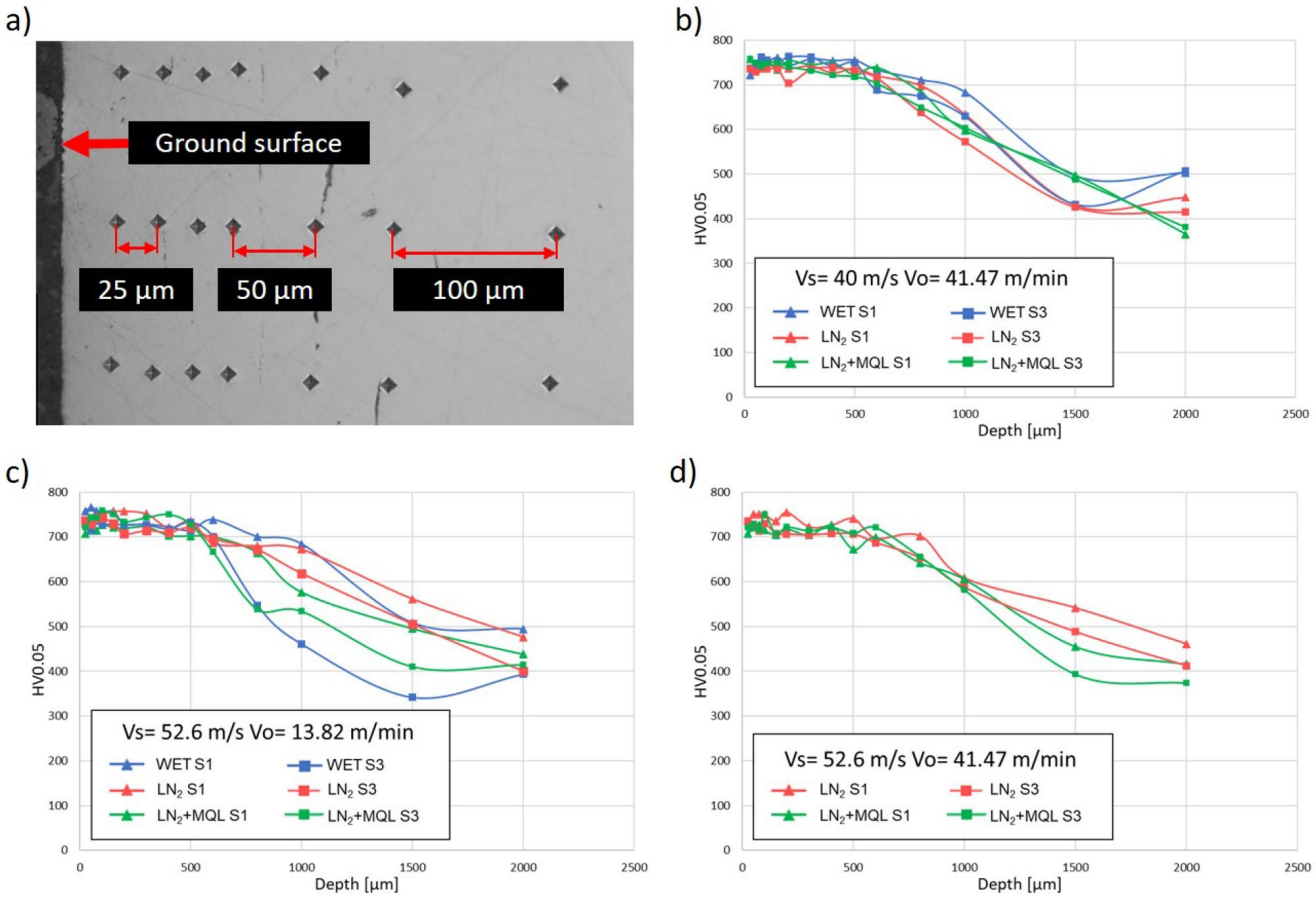
Microhardness profiles HV_0.05_ for the best machining conditions. *(***a**) Hardness test depths on the cross-section, *(***b**) Vs = 40 m/s, Vo = 41.47 m/min, *(***c**) Vs = 52.6 m/s, Vo = 13.82 m/min, and *(***d**) Vs = 52.6 m/s, Vo = 41.47 m/min.

The HV_0.05_ microhardness profiles of the three best machining conditions are illustrated in Fig. 6. Slight differences can be observed in the decrease of the curves for depths close to 500 µm. However, it could also be due to measurement uncertainty because, at these depths, only the carburized treatment influences the material, and it would have to be the same. In contrast, according to “Microstructural analysis” section, the maximum depths of the thermally affected layer were close to 25 µm. If the microhardness profiles of Fig. 6 are analyzed at these depths, the similarity between these suggests that no significant differences can be evidenced in the use of cryogenic fluids (LN_2_, LN_2_+MQL) and the conventional flood system (WET). Additionally, it seems that no grinding burns were produced on the material surface because microhardness profiles did not present decrements near the ground surface.

### Residual stresses analysis

The residual stress on ground surfaces can be affected by several mechanisms^42^. (i) Typically, in the grinding process, the rubbing and plowing of grits on the material surface induces plastic deformation, removing small swarf due to the mechanical abrasion that produces compressive surface residual stresses. (ii) Plastic deformation induced by thermally effects product of the energy dissipation of the process. This mechanism is important to consider because it provokes expansion/contraction of the material surface that commonly produces tensile stress at the surface^40,42^. (iii) In some cases, depending on the microstructural structure and the thermal properties of the material, the thermal effect and the forces induced in the process can produce a microstructural phase transformation in the material. Due to this phase transformation, the material can present both compression and tensile surface residual stress^20,40,43,44^.

**Figure 7:**
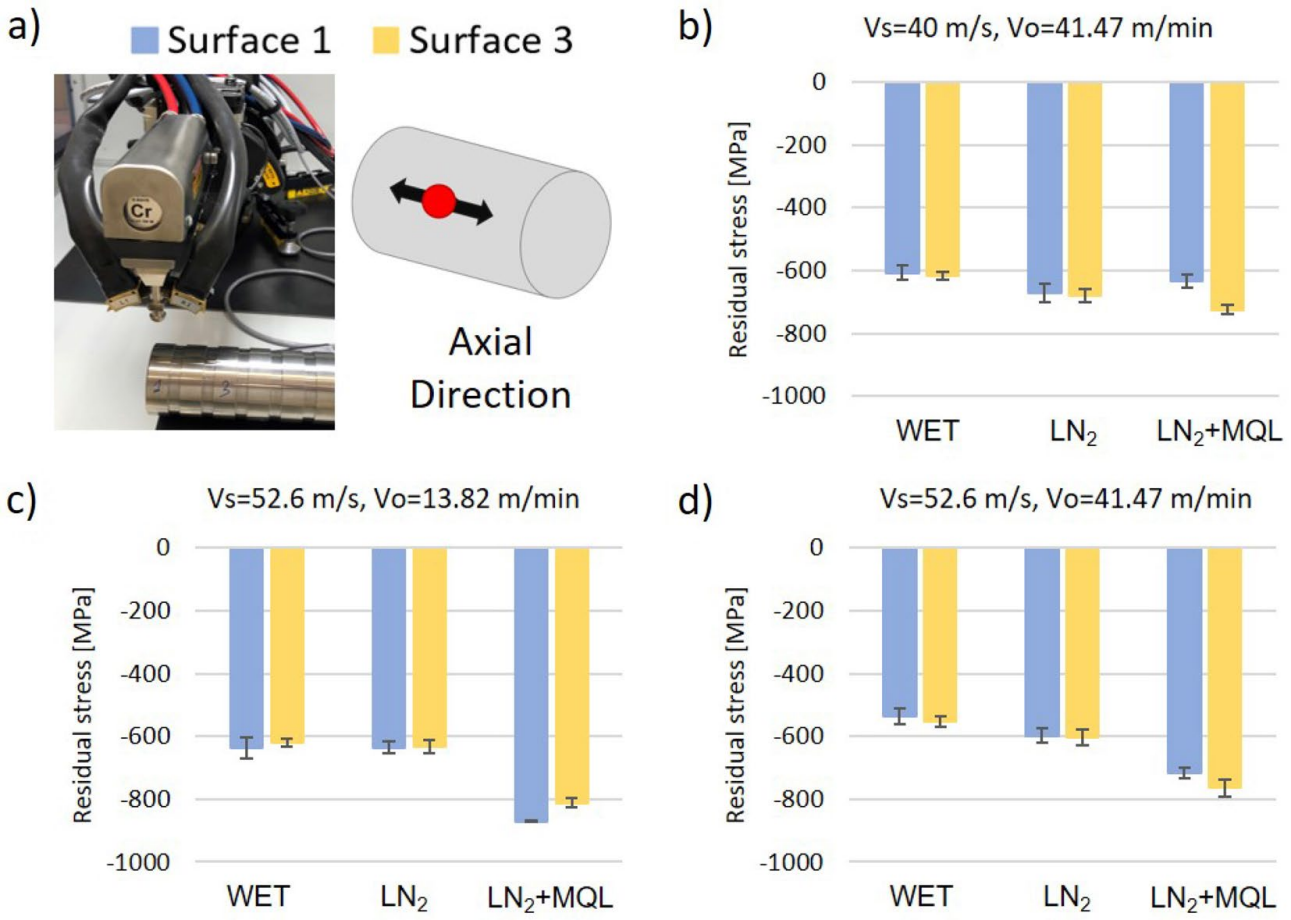
Axial residual stress on the first and third ground surfaces with best grinding conditions. (**a**) Scheme of measures, (**b**) Vs = 40 m/s, Vo = 41.47 m/min, (**c**) Vs = 52.6 m/s, Vo = 13.82 m/min, and (**d**) Vs = 52.6 m/s, Vo = 41.47 m/min.

**Figure 8:**
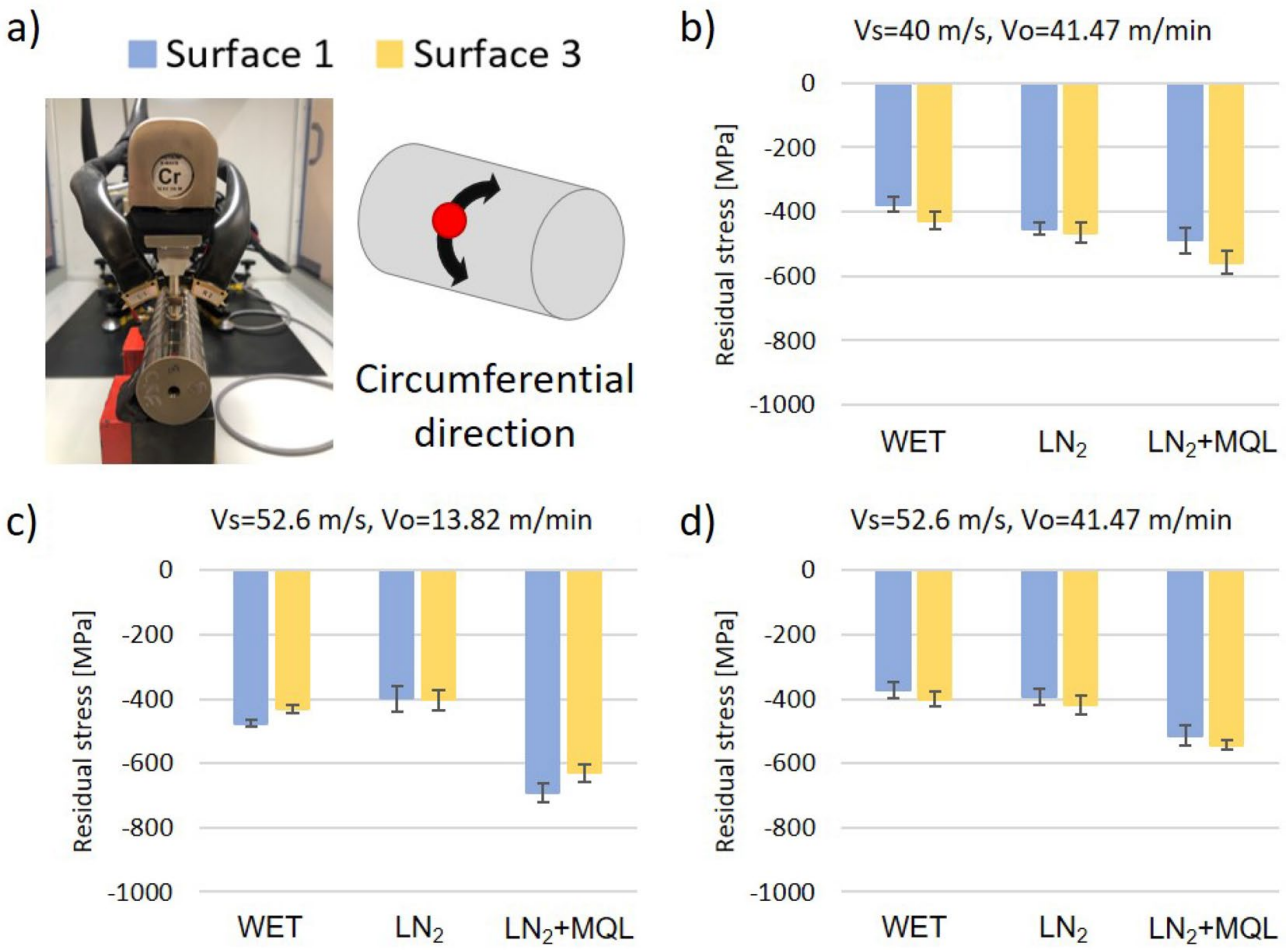
Circumferential residual stress on the first and third ground surfaces with best grinding conditions. (**a**) Scheme of measures, (**b**) Vs = 40 m/s, Vo = 41.47 m/min, (**c**) Vs = 52.6 m/s, Vo = 13.82 m/min, and (**d**) Vs = 52.6 m/s, Vo = 41.47 m/min.

The circumferential and axial surface residual stresses were measured for the three best grinding conditions with the X-ray diffraction technique (see Figs. 7a, 8a). According to these results, all tested surfaces showed compressive stresses. This predominant behavior was in accordance with the first pattern found in the literature for mechanical abrasion^42^. Moreover, the second pattern due to thermal effects might be minimized because the ground material was steel with good thermal conductivity, and all ground surfaces kept good cooling during the process, which reduces thermal gradients. Furthermore, as the authors explained in the microstructural analysis of “Microstructural analysis” section, the material was carburized before the tests, obtaining a martensitic structure; therefore, the third pattern found in the literature did not apply for this work because as this work demonstrated in the microstructural analysis, the material did not suffer a phase transformation.

Figure 7 illustrates the axial surface residual stresses, in which compressive values between 538 and 870 MPa are shown. However, residual stresses in the circumferential direction were lower. According to Fig. 8, circumferential compressive residual stresses are in the range of 374 to 691 MPa. Independent of the cooling fluid utilized, the highest compressive residual stresses in both directions, axial and circumferential, were obtained by the machining condition (*V*s = 52.6 m/s and *V*o = 13.82 m/min). This trend suggests that the compressive residual stresses increase when the grinding wheel speed is more aggressive due to the rise in abrasive forces since it seems to be the dominant mechanism in the generation of residual stresses (Figs. 7c, 8c).

Also, the accumulated ground volume of the grinding wheel presented effects on the residual stresses. As this work explains in “Roughness surface analysis” section, for lower workpiece speed, the stability of the process increased, generating a reduction in roughness variance between subsequent ground surfaces. The same trend was observed in the analysis of residual stresses; the ground surfaces generated by the middle wheel speed (*V*s = 52.6 m/s) and low workpiece speed (*V*o = 13.82 m/min) showed a decreasing trend between the first and third ground surfaces for all coolant fluids (Fig. 7c, 8c). However, this was not the case of the other machining conditions (*V*s = 40 m/s and *V*o = 41.47 m/min, and *V*s = 52.6 m/s and *V*o = 41.47 m/min), in which increasing trends in the residual stress were found between surface 1 and surface 3. This result supports the hypothesis that process stability is essential to produce good surface integrity by reducing the variance in the process.

The application of cryogenic fluids on the plunge grinding process reflects additional features in the generation of residual stresses. Figures 7 and 8 show that the machined surfaces with LN_2_+MQL generated the highest compressive stresses on the material for all tested conditions. In contrast, the differences between LN_2_ and WET coolants were not apparent. The application of LN_2_ and WET on ground surfaces generated by a medium wheel speed (*V*s = 52.6 m/s) and low workpiece speed (*V*o = 13.82 m/min) did not present differences in residual stresses. Nevertheless, there were slight differences in magnitudes for the other machining condition since when the LN_2_ system was applied, compressive stresses were higher. These remarks suggest that the thermal effect on the generation of residual stresses was minimized when using cryogenic coolants compared to the WET cooling systems.

Finally, thanks to the complete surface integrity analysis done in this work, it has been possible to demonstrate that under optimum conditions of the grinding wheel (after dressing the wheel), the cylindrical cryogenic plunge grinding (LN_2_ and LN_2_+ MQL) can be a feasible solution to diminish/avoid conventional coolant/lubricants fluids that pollute the environment and may cause injuries to machine operators. When the process stability was considered in the analysis by machining several surfaces before dressing the wheel, the experiments demonstrated a slight increase in surface roughness for all tested coolant systems. This finding suggests that wheel wear should be controlled, and dressing should be applied periodically. The condition Vs = 52.6 m/s and Vo = 13.82 m/min obtained the best combination state: lower surface roughness (Ra ≈ 0.17 to 0.25 µm) with the highest stability (Ra roughness slightly varied from the first surface to the last ground surface) for all the tested cooling systems. This selected condition did not present significant defects in the surface and subsurface of the material for the tested coolant/lubricant systems, and the microhardness profiles did not expose any differences. Ultimately, the residual stress analysis showed that the LN_2_ system at least produces the same compressive surface residual stresses as the WET system, and the LN_2_+MQL system produces higher compressive surface residual stresses than the other systems. This response in the material surface may increase mechanical and fatigue properties that should be studied in future research.

## Conclusions

The cryogenic plunge grinding was investigated using LN_2_ and LN_2_+MQL. The results of this process were compared to plunge grinding using a conventional emulsion. The analysis was made in terms of the final surface integrity of the workpiece, including surface roughness, microstructure defects, hardness profiles, and surface residual stresses. Based on the results obtained in this work, the following conclusions can be drawn:

- The results obtained in this work demonstrate that the uses of LN_2_ and LN_2_+MQL as coolant/lubricant systems can replace conventional flood coolant with very similar values in terms of surface roughness and surface integrity, even after several subsequently ground surfaces. The proposed innovative grinding approaches exposed will help reducing the consumption of flood contaminant coolants, improving the advances in cryo-coolant/lubricant systems to protect the environment.
- Analysis of the microstructural subsurface does not indicate significant defects or microstructural changes due to the application of cryogenic fluids in the plunge grinding process of carburized 27MnCr5 steel. Furthermore, the application of cryogenic coolants like LN_2_ and LN_2_+MQL present fewer defects than WET samples. Additionally, the microhardness profiles generated in the material due to the use of cryogenic coolants in the plunge grinding process are similar to those obtained when using conventional coolant.
- In all tested conditions, compressive residual stresses were generated because of the predominance of the abrasive mechanism over the thermal effect. Interestingly, the application of cryogenic coolant in the plunge cylindrical grinding shows an increment of the compressive surface residual stresses of the 27CrMn5. LN_2_ presents an increase among 0–20% of compressive stresses compared to WET conditions, and the LN_2_+MQL presents an increase among 4–45% of compressive residual stresses compared to WET conditions.

## Data Availability

The dataset generated and/or analyzed during the current study are available in Mendeley: Abedrabbo, Anibal Faruk (2021), “Cryogenic plunge cylindrical grinding Dataset”, Mendeley Data, V1, https://doi.org/10.17632/4n2nfx6cx9.1.
